# Exposure to the Neurotoxic Dinoflagellate, *Alexandrium catenella*, Induces Apoptosis of the Hemocytes of the Oyster, *Crassostrea gigas*

**DOI:** 10.3390/md11124799

**Published:** 2013-12-02

**Authors:** Walid Medhioub, Simon Ramondenc, Audrey Sophie Vanhove, Agnes Vergnes, Estelle Masseret, Veronique Savar, Zouher Amzil, Mohamed Laabir, Jean Luc Rolland

**Affiliations:** 1 Institut National des Sciences et Technologies de la Mer, Laboratoire Milieu Marin, 28 rue du 2 mars 1934, Salammbô 2025, Tunisia; E-Mail: medhwalid@yahoo.fr; 2 Institut Français de Recherche pour l'Exploitation de la Mer, Centre National de la Recherche Scientifique, Université de Montpellier 2, Université de Montpellier 1, Institut de la Recherche pour le Développement, Unité Mixte de Recherche 5119 “Ecologie des Systèmes Marins Côtiers”, Place Eugene Bataillon, CC93, Montpellier cedex 5, 34095, France; E-Mails: simon.ramondenc@gmail.com (S.R.); audrey. vanhove@ifremer.fr (A.S.V.); agnes.vergnes@ifremer.fr (A.V.); estelle.masseret@univ-montp2.fr (E.M.); mohamed.laabir@univ-montp2.fr (M.L.); 3 Institut Français de Recherche pour l'Exploitation de la Mer, Laboratoire Environnement, Microbiologie et Phycotoxines, Rue de l’Ile d’Yeu BP 21105, Nantes CEDEX 3 44311, France; E-Mails: veronique.savar@ifremer.fr (V.S.); zouher.amzil@ifremer.fr (Z.A.)

**Keywords:** shellfish, toxins, apoptosis, gene expression

## Abstract

This study assessed the apoptotic process occurring in the hemocytes of the Pacific oyster, *Crassostrea gigas*, exposed to *Alexandrium catenella*, a paralytic shellfish toxins (PSTs) producer. Oysters were experimentally exposed during 48 h to the toxic algae. PSTs accumulation, the expression of 12 key apoptotic-related genes, as well as the variation of the number of hemocytes in apoptosis was measured at time intervals during the experiment. Results show a significant increase of the number of hemocytes in apoptosis after 29 h of exposure. Two pro-apoptotic genes (Bax and Bax-like) implicated in the mitochondrial pathway were significantly upregulated at 21 h followed by the overexpression of two caspase executor genes (caspase-3 and caspase-7) at 29 h, suggesting that the intrinsic pathway was activated. No modulation of the expression of genes implicated in the cell signaling Fas-Associated protein with Death Domain (FADD) and initiation-phase (caspase-2) was observed, suggesting that only the extrinsic pathway was not activated. Moreover, the clear time-dependent upregulation of five (Bcl2, BI-1, IAP1, IAP7B and Hsp70) inhibitors of apoptosis-related genes associated with the return to the initial number of hemocytes in apoptosis at 48 h of exposure suggests the involvement of strong regulatory mechanisms of apoptosis occurring in the hemocytes of the Pacific oyster.

## 1. Introduction

Apoptosis or type I programmed cell death was reported to play an important role in organism immunity, especially in mollusks [1,2]. Based on the recent generation of Expressed Sequences Tags (EST) in the Pacific oyster, *Crassostrea gigas*, the basic genes and domains related to apoptosis-associated proteins were demonstrated to be conserved [3]. Apoptosis is initiated by either extracellular or intracellular signals. Extracellular signals activate the extrinsic pathway (receptor-mediated) through death receptors. These activated receptors recruit the cell signaling Fas-Associated protein with Death Domain (FADD), forming the death-inducing signaling complex (DISC), inducing the activation of initiator cysteine proteases of the caspase family. The intrinsic apoptotic pathway (mitochondrial) is activated in response to cytotoxic stimuli or environmental stressors. In vertebrates when stressed, mitochondria become permeable and release cytochrome C into the cytosol [4]. Cytochrome C induces the formation of the apoptosome complex (Apaf-1/cytochrome c/caspase-9), then activates executor caspases, which play a central role in the execution phase of cell apoptosis [5]. B-cell lymphoma 2 (Bcl-2) family proteins regulate this process by releasing apoptotic signals from the mitochondria [6,7,8,9]. Members of this family are conserved in invertebrate [10]. This family is composed of pro- and anti-apoptotic members. Anti-apoptotic Bcl-2 members display sequence homology in four α-helical domains, called BH1–BH4 [10,11]. Pro-apoptosis can be further subdivided into more fully conserved, “multi-domain” members with homology in the BH1–BH3 domains and BH3-only Bcl-2 family proteins. The cell death process is also regulated by inhibitors of caspase, inhibitors of apoptosis (IAPs) [6] and heat shock proteins (Hsps) [12,13,14]. 

PSPs (paralytic shellfish poisons) are neurotoxins naturally produced by cyanobacteria and a number of toxic dinoflagellate species, such as *Alexandrium catenella*, *Gymnodinium catenatum* and *Pyrodinium bahamense* [15,16]. These toxins were demonstrated to be highly accumulated in oysters that feed on dinoflagellates [17]. Globally, such paralytic shellfish toxin (PST) producing species negatively affect the clearance rate, the feeding and digestive capacity of exploited bivalves [18,19,20,21,22]. However, no direct lethal effect on oyster has been shown. Saxitoxin and its analogs are potent Na^+^ channel blockers in vertebrate cells and mitochondria and have negative effects on the cellular integrity of mollusk cells [23,24,25]. 

The aim of the present study was to investigate if exposure to a dense concentration of the toxic dinoflagellate, *A. catenella*, induces the apoptosis of the effective cells involved in the immune responses of oyster, the hemocytes. Oysters were experimentally exposed during 48 h to a toxic strain of *A. catenella* or a strain of *A. tamarense*, a non-producer of PSTs, considered as the control. PST accumulation was measured in oyster tissues. Temporal expressions of putative apoptotic-related genes previously identified from the 210,895 ESTs from *C. gigas* reported in the National Center of Biotechnology Information (NCBI) database as of 1 May 2013, were determined. Additionally, evolutions of the number of apoptotic cells were determined by microscopy during the experiment. 

## 2. Results and Discussion

### 2.1. PSP Accumulation in Oyster Tissues

**Figure 1:**
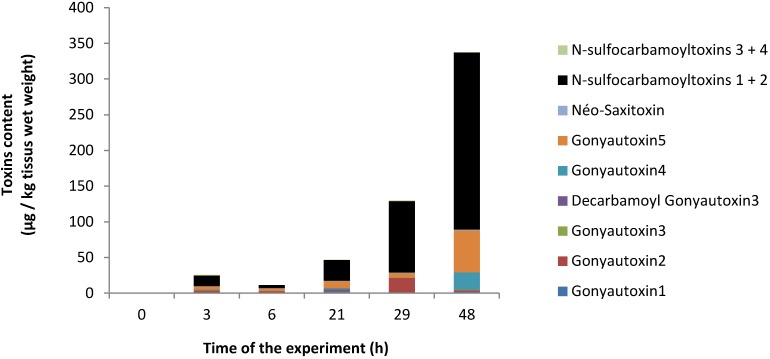
Evolution of the paralytic shellfish poison (PSP)-toxin content (µg/kg wet weight) in *Crassostrea gigas* exposed to *Alexandrium catenella*; the bar charts represent (in %) the temporal toxin.

The *Alexandrium catenella* (ACT03) strain contained 5.3 ± 0.4 pg toxins/cell. The following toxins were found in decreasing concentrations: *N*-sulfocarbamoyltoxins 1 + 2 (51%), Gonyautoxins 5 (35%), Gonyautoxins 4 (12%), Gonyautoxins 1 (1%) and neo-Saxitoxin (1%) with *N*-sulfocarbamoyltoxins 4, Gonyautoxins 3, Saxitoxin and decarbamoyl saxitoxin present as trace amounts (not show). During the 48 h of the experiment, PSP toxins accumulated in oysters (Figure 1). The toxicity level reached 10 (2.2), 40 (2.07), 40 (7.4), 130 (13.6) and 360 (53.2) µg/kg tissue wet weight (µg Saxitoxin diHCl equivalent/kg wet weight) at three, six, 21, 29 and 48 h, respectively. Due to the small amount of cells available for the analysis, the PST accumulation in hemocytes was not determined. 

The PST concentration in oyster after 48 h of exposure reaches 360 (53.2) µg/kg tissue wet weight (µg STX diHCl equivalent/kg wet weight). Such a level of toxicity was low, but close to that generally found in oyster in the environment. Since 1988, oysters cultivated in the French Mediterranean Thau lagoon were frequently contaminated by PSP toxins during spring and/or autumn, but rarely did there contamination exceeded the sanitary threshold (800 µg Saxitoxin diHCl equivalent/kg tissue wet weight) excepted in 2001 and 2003 [26].

### 2.2. Level of Hemocytes in Apoptosis

**Figure 2:**
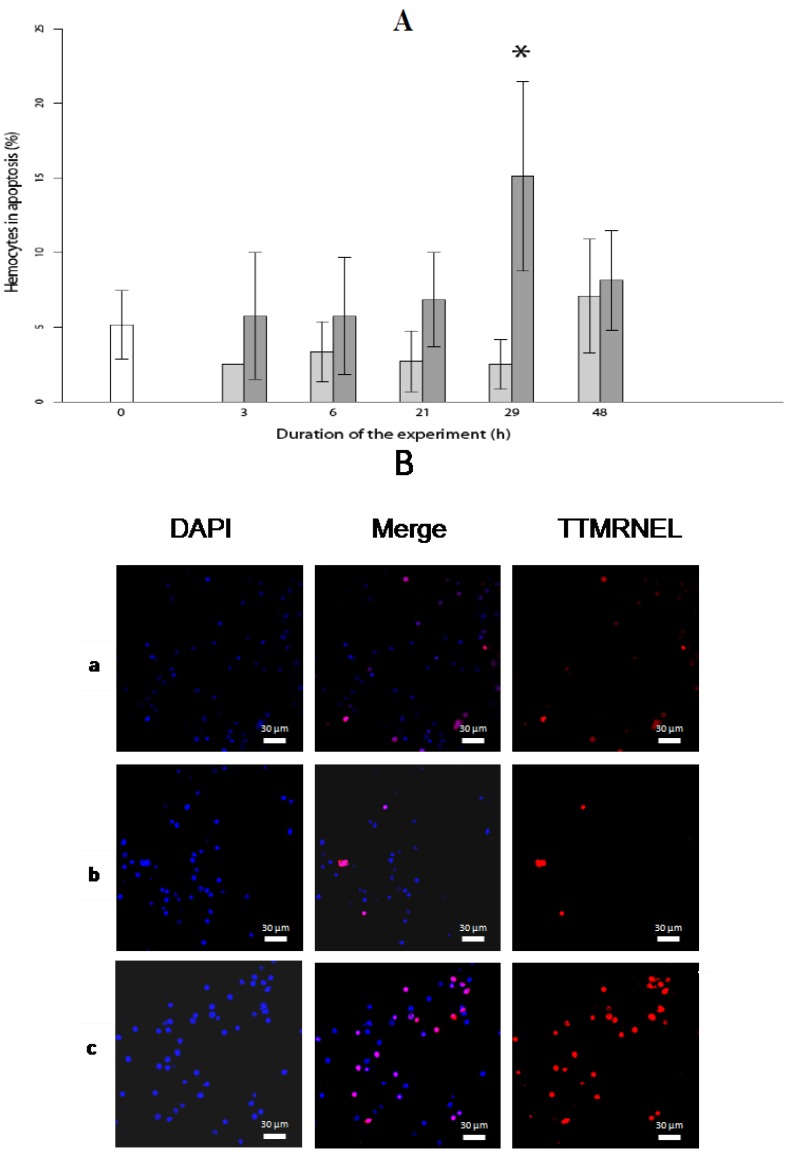
Evaluation of the number of hemocytes in apoptosis in oysters exposed for 48 h to *A. catenella*, (**A**) Percentage of cells in apoptosis. Oyster not exposed (white), exposed to *Alexandrium tamarense* (grey) or to *Alexandrium catenella* (dark grey), * (ANOVA, *p* < 0.01); (**B**) Hemocyte observations of oyster non-exposed (a), exposed for 29 h to *A. tamarense* (b) or to *A. catenella* (c) after Terminal deoxynucleotidyl transferase TetraMethylRhodamine Nick End Labelling (TTMRNEL) staining (nuclei are stained in blue and apoptotic cells in red).

Exposure of oysters to *A. catenella* increased the number of nuclear degradations at 29 h (ANOVA, *p <* 0.01), which coincided with a toxins concentration of 130 µg/kg oyster wet tissue (Figure 2). The hallmark of apoptosis is DNA degradation, which, in the early stages, is selective to the internucleosomal DNA linker regions. Many chemical agents have been shown to induce apoptosis in mollusks. Among these agents, heavy metals have been well documented in terms of their toxicity on ionic channels and the ability to bioaccumulate in the tissues [27]. For example, cadmium has been demonstrated to inhibit GABA-activated ion currents by increasing intracellular calcium levels in snail neurons [28] and to induce apoptosis in the hemocytes of the oyster, *Crassostrea virginica*, through a mitochondria/caspase-independent pathway [29]. Like heavy metals, saxitoxin and its analogs, all potent Na^+^ channel blockers, may be responsible for the apoptosis observed here.

Surprisingly, after 48 h of exposure, while the concentration still increased to reach 0.36 µg/kg wet weights, no significant difference in the number of hemocytes in apoptosis was observed in comparison to the control (Figure 2). This suggests the implementation of an efficient regulatory mechanism to control apoptosis.

### 2.3. Temporal Expression of the Genes Related to Apoptotic Processes

The expression level of putative apoptotic-related genes was evaluated in hemocytes of *C. gigas* exposed to toxic *A. catenella* or to the non-toxic *A. tamarense* at zero, three, six, 21, 29 and 48 h after the beginning of the experiment. The genes selected are involved in the intrinsic pathway (Bax, Bax-like, Bcl2, BI-1), cell signaling (FADD), initiation-phase (caspase-2) and execution phase of cell apoptosis (caspase-3 and caspase-7). Other key genes associated with the regulation of the apoptosis system, executor caspase inhibitors (IAP1 and IAP-7B) and stress proteins (Hsp70 and Hsp27), were also analyzed. 

#### 2.3.1. Expression of Apoptosis-Related Genes

**Figure 3:**
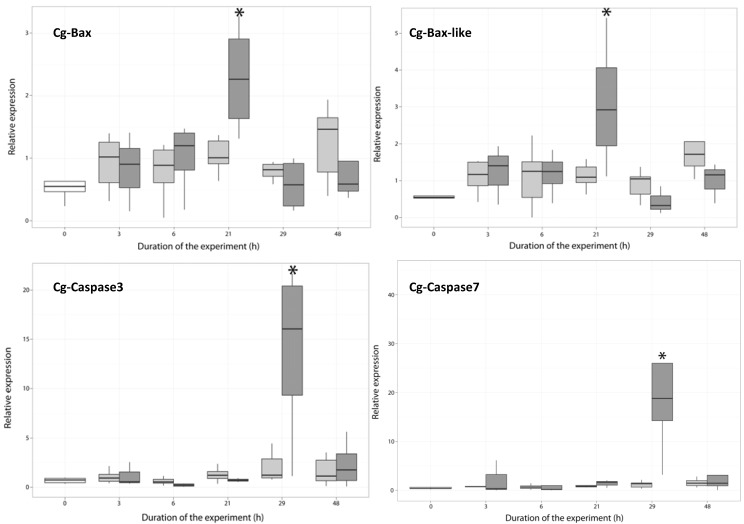
Expression of cg-Bax, cg-Bax-like, cg-caspase-3 and cg-caspase-7 in the hemocyte of *Crassostrea gigas* not exposed (time 0, white), exposed to *Alexandrium tamarense* (grey) or to *Alexandrium catenella* (dark grey). * (ANOVA, *p* < 0.01).

The deduced amino-acid sequence of cg-Bax and cg-Bax-like display more than 40% identity with apoptotic Bax family members in three-helical domains, called BH1–BH3. Interestingly, cg-Bax-likeshows more than 97% identity with the apoptosis regulator, Bcl-2 (EKC30556), and the Bcl-2-like protein 1 (EKC30554) and 72% identity with the Bcl-2-associated X protein from the mussel, *Mytilus galloprovincialis* (AGK88247.1), but has no BH4 domain. Results showed that Bax transcripts were significantly overexpressed (ANOVA, *p* < 0.01) at 21 h in oysters exposed to *A. atenella* (Figure 3), whereas these genes were not modulated in the hemocytes of oysters exposed to *A. tamarense*. In mammals, Bax members play a central role in the induction of the mitochondrial apoptosis pathway. When cells are exposed to various apoptotic stimuli, the proteins are overexpressed [30] and translocated into the mitochondria [31,32]. In the mitochondria, Bax forms oligomers in the outer membrane, permeabilizing the membrane and promoting the release of apoptotic factors, such as cytochrome c, into the cytosol [33,34].

Cg-caspase-3 protein **(**EKC30354.1) and Cg-caspase-7 protein (EKC34323.1) display high identity with members of the executioner caspase (cysteine aspartate protease) family of proteins. Interestingly, *C. gigas* translated caspase-7 partial coding sequence (CU988427.1) and caspase-1 complete coding sequence (HQ425703.1) mRNA sequences display high identity with cg-caspase-3 **(**EKC30354.1). The *C. gigas* translated caspase-3/-7complete cds (HQ425703.1) mRNA sequences display high identity with the cg-caspase-7 (EKC34323.1). Compared to the control, the level of cg-caspase-3 and cg-caspase-7 transcript increased significantly at 29 h (Figure 3) in the hemocytes of oysters fed the toxic *A. catenella* (*p* < 0.05), but was not modulated in the hemocytes of oysters fed with the non-toxic *A. tamarense*. In vertebrates, caspases play a central role in the execution-phase of cell apoptosis [35,36,37].

FADD and caspase-2 gene expression was not modulated in the hemocytes of *C. gigas* exposed to toxic or nontoxic *Alexandrium species* (not shown). In response to cytotoxic stimuli, DNA damage or environmental stressors, the signaling of the vertebrate extrinsic pathway begins with death receptor activation. It requires the interactions of their death domains and the downstream adapter, FADD [38]. Then, caspase-2 is activated, and apoptosis occurs [39]. These two genes were shown to be upregulated in *C. gigas* infected with *Vibrio anguillarum* [3]. This result suggests that the extrinsic pathway of apoptosis was not activated. 

#### 2.3.2. Expression of Anti-Apoptosis-Related Genes

**Figure 4:**
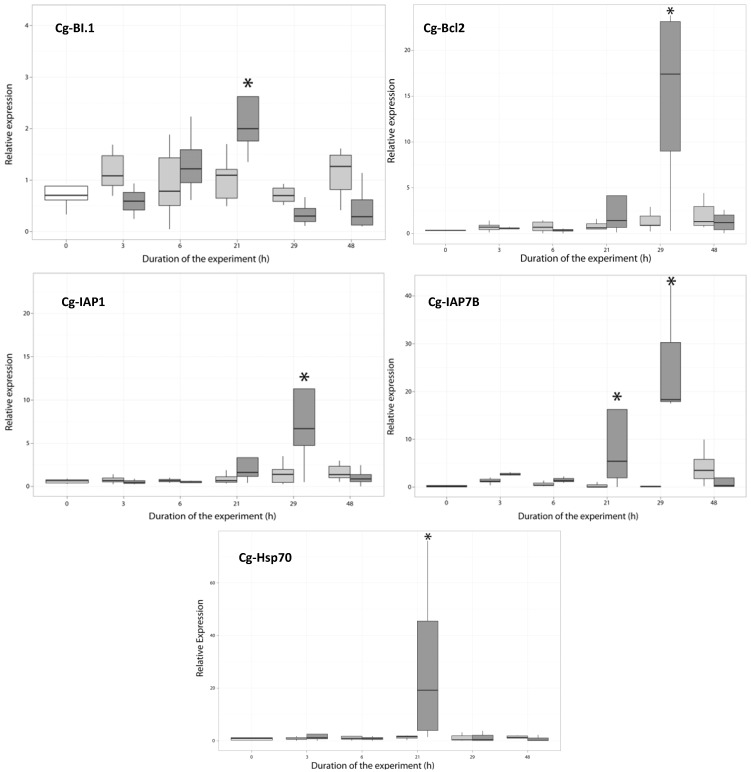
Relative expression of cg-BI.1, Cg-Bcl2, Cg-IAP1, Cg-IAP7B and cg-Hsp70 in the hemocytes of *Crassostrea gigas* not exposed (time 0, white), exposed to *Alexandrium tamarense* (grey) or to *Alexandrium catenella* (dark grey). * (ANOVA, *p* < 0.01).

The cg-*Bcl2* protein displays more than 30% identity with anti-apoptotic Bcl-2 family members in four-helical domains, called BH1–BH4. This transcript was significantly overexpressed at 29 h (ANOVA, *p* < 0.01) in oysters exposed to *A. catenella* (Figure 4). Bcl2 members are present in the mitochondrial membrane and are able to form a heterodimer with Bax [40]. The observed time-dependent regulated expression of this anti-apoptosis-related gene suggested the involvement of regulatory mechanisms to control apoptosis.

The transmembrane Bax inhibitor (cg-BI.1) displays sequence homology with Golgi Anti-Apoptotic Protein (GAAP) or the transmembrane Bax inhibitor motif containing the 4.Cg-BI.1 transcript was significantly overexpressed at 21 h (ANOVA, *p* < 0.01) in oysters fed with *A. catenella* (Figure 4). Moreover, the upregulation at 21 h of cg-BI.1 was followed by the downregulation of cg-Bax and the upregulation of cg-Bcl2 at 29 h (Figure 3). In mammalian cells, the transmembrane Bax inhibitor was demonstrated to inhibit the decrease in the mitochondrial membrane potential, by either stimulating the anti-apoptotic function of Bcl-2 or inhibiting the pro-apoptotic effect of Bax [41,42]. Another study showed that BI.1 interacts with Bcl2 to increase the acidity of the cytoplasm, promoting ATP production by mitochondria, which contributes toward maintaining a neutral Ph in the cytosol [43].

Inhibitors of caspases (IAPs) were known to contribute significantly to cell death regulation by blocking the catalytic site of the caspase executor [44]. In the genome of *C. gigas*, 48 genes coding IAPs were identified [45], when only three and seven genes found in human and in sea urchin, respectively. In this work, we analyze the expression of two of them, because their sequences are available in GenBank. The two IAP genes display 25% to 44% identity with IAP family members in two baculovirus inhibitor of apoptosis protein repeat (BIR) domains. IAP1 and IAP7B display 43% and 44% identity with the IAP of the giant panda, *Ailuropoda melanoleuca* (XP002922057), respectively. In this experiment, the transcript levels of cg-IAP1 and cg-IAP7B proteins increased from 21 h to reach their highest levels at 29 h (*p* < 0.01) in the hemocytes of oysters exposed to *A. catenella*, whereas those genes were not modulated in the hemocytes of oysters exposed to *A. tamarense* (Figure 4). IAPs were overexpressed after the expression of Bax (Figure 3). This result is in accordance with the work of Wei and Devreaux (1999), who demonstrated that the overexpression of the apoptotic inhibitor in human was induced by Bax or other Bcl2 apoptosis family members [6,46].

Cg-Hsp70 was significantly overexpressed at 21 h (ANOVA, *p* < 0.01) in oysters exposed to *A. catenella* (Figure 4), and the Cg-Hsp27 transcript was not modulated (not show). The induction of chaperones has already been demonstrated in many organisms [47,48,49,50] as a response to stress. Moreover, Hsp70 and Hsp27 have been demonstrated to be anti-apoptotic, interacting with the components of the apoptotic pathways [12,13,14,51]. The parasite, *Perkinsus marinus*, employs heat shock proteins as part of its adaptive survival repertoire in the oyster, *C. Virginica* [52,53]. Interestingly, in *C. gigas*, the expression of Hsp70 was demonstrated to decrease when the oyster is exposed to cadmium [54]. Furthermore, cadmium is known to induce apoptosis in oyster hemocytes by disturbing the cellular energy balance [29]. Together with the result obtained in the present study, the upregulation of cg-Hsp70 could be associated with the ability of *C. gigas* to control apoptosis.

Nevertheless, the clear upregulation of the expression of five of six inhibitors of apoptosis-related genes tested suggests the involvement of strong regulatory mechanisms to control the apoptosis occurring in the hemocytes of the Pacific oyster.

## 3. Experimental Section

### 3.1. Oysters and Microalgae

Adult Pacific oysters (*Crassostrea gigas*) were collected in November, 2011, from an oyster farm in the Thau lagoon (Masson, Société A Responsabilité Limitée, Languedoc-Roussillon, France) during periods when blooms did not occur. The average total oyster fresh weight was 13.0 ± 2.9 g (average ± SD); the average digestive gland weight was 1.8 ± 0.5 g, and the average shell length was 11.0 ± 1.0 cm. Before the experiments, oysters were exposed to a continuous flow of filtered (10 µm) Mediterranean seawater, maintained in partial starvation, having only bacteria and nanoplankton to feed, at a constant temperature of 20 ± 1 °C for two weeks for acclimatization.

The experiments were carried out with a toxic *A. catenella* (ACT03 strain) and a non-producer of PSTs, *A. tamarense* (ATT07 strain), isolated from the Thau lagoon in 2003 and 2007, respectively. The ENSW (enriched natural sea water) [55] culture medium used was characterized by a salinity of 35 practical salinity units (PSU). The two dinoflagellate species were cultivated in batch cultures and were grown at 20 ± 1 °C, under cool-white fluorescent illumination (100 µmoles photons/m^2^/s) and a 12 h:12 h light:dark cycle. For the feeding experiments, we used algae in their exponential growth phase.

### 3.2. Experimental Exposures

Two independent experimental exposures were carried out. For each experiment, after two weeks of acclimatization, 180 oysters were randomly placed into six tanks (30 individuals per tank) containing 10 L of filtered (0.2 µm) seawater. The experiments were conducted at a constant temperature of 20 ± 1 °C. Cells of *A. catenella* (two tanks) or *A. tamarense* (two tanks) were added into tank water regularly to maintain cell concentrations (1 × 10^6^ cells/L) corresponding to the *in situ* bloom in Thau lagoon [56]. In two control thanks, oysters were incubated in filtered (0.2 µm) sea water without algae. The mean concentrations in the tank water of the experiments for toxic *A. catenella* and non-toxic *A. tamarense* were (1.35 ± 0.02) × 10^6^ cells/L and (2.20 ± 0.23) × 10^6^ cells/L, respectively. Fresh cells were regularly added at 3, 6, 21 and 29 h to approach the initial cell concentrations. However, during the 48-h experiment, the concentrations in tank water ranged between 1 × 10^6^ cells/L and 2.5 × 10^6^ cells/L. To estimate the concentration of cells in tanks during the experiment, triplicates of 1 mL of water were collected, and cells were fixed with Formalin (2%), then counted in a Nageotte counting chamber using a photonic microscope.

### 3.3. Tissue Sampling

Hemolymph from 5 oysters randomly taken from tanks (containing 30 individuals each) was collected at time 0 (control), 3, 6, 21, 29 and 48 h for analysis. The remaining tissues were pooled and stored at −20 °C until the toxin extraction was performed.

### 3.4. Chemical Analysis of PSP Toxin by Liquid Chromatography/Fluorescence Detection (LC/FD)

One milliliter of 0.1 N acetic acid was added to the pooled tissues, and the samples were frozen at −20 °C until the extraction and analysis were performed. To release the toxins, the samples were sonicated for 5 min in a water bath three times and centrifuged at 17,000× *g* for 10 min at 4 °C. The supernatants were used for the subsequent LC/FD PSP toxin analyses, using the method of Oshima [57]. The toxins were separated by reverse chromatography using a C_8_ column (5 µm Develosil, 4.6 mm i.d. × 250 mm) with a flow rate of 0.8 mL/min. The eluent pH and/or column temperature were calibrated to optimize the separation of some gonyautoxins (dc‑DTX3/B1/dc-GTX-2). The toxins were quantified using certified standards provided by the National Research Council of Canada (Halifax, Canada). B2 and C2-toxins were detected and quantified indirectly after acid hydrolysis (HCl 0.4 N at 97 °C for 5 min). The toxin concentration (µg/g) was converted into µg STX equiv/Kg wet weight of tissues using the conversion factors determined by Oshima [58].

Triplicates of 10 mL batch cultures (cell concentration ≥ 10^7^ cells/L) were taken during the exponential growth phases of the cultivated dinoflagellates. After centrifugation (3000× *g*, 8 min, 4 °C), the cells were suspended in 1 mL of 0.1 N acetic acid and frozen at −20 °C. The extraction and toxin analyses were performed as explained above.

### 3.5. Expression Analysis of Putative Apoptotic-Related Genes

The expression levels of putative apoptotic-related genes were measured in hemocytes of *C. gigas* fed with *A. catenella* or *A. tamarense* at 0, 3, 6, 21, 29 and 48 h after the beginning of the experiment. Hemocytes from one milliliter of hemolymph were collected and placed in 0.5 mL of Trizol buffer and conserved at −20 °C. Total RNA was isolated from the oyster hemocytes using the standard Trizol method (Invitrogen Life Technologies SAS, Saint Aubin, France), then treated with DNAse (Invitrogen) to eliminate the contamination of genomic DNA. After sodium acetate precipitation, the quantity and quality of total RNA were determined using a NanoDrop spectrophotometer (NanoDrop Technologies, Wilmington, DE, USA) and agarose gel electrophoresis, respectively. Following heat denaturing (70 °C for 5 min), reverse transcription was performed using 0.1 µg of hemocyte RNA prepared with 50 ng/µL oligo-(dT)_12mer−18mer_ in a 20-µL reaction volume containing 1 mM dNTPs, 1 unit/µL of RNAseOUT and 200 units/µL Moloney Murine Leukemia Virus Reverse Transcriptase (M-MLV RT) in reverse transcriptase buffer, according to the manufacturer’s instructions (Invitrogen Life Technologies SAS, Saint Aubin, France).

The primer pairs used to quantify the expression level of apoptotic-related genes were designed according to the sequence available in Gene-Bank. The expression of the ribosomal protein, F40, was used as the housekeeping gene control. All sequences of primers used for the amplification are shown in Table 1. Real-time PCR amplifications were performed in the Light Cycler 480 (Roche). In short, the following components were mixed to the indicated end-concentration: 5 mM MgCl_2_, 0.5 µM of each primer, 2.5 μL of reaction mix (Light Cycler^®^ 480 SYBR^®^ Green I Master mix) in a final volume of 5 μL. Reverse transcribed RNA (1 μL) diluted 1/10 was added as the PCR template to the Light-Cycler master mix, and the following run protocol was used: initial denaturing at 95 °C for 5 min; 95 °C for 10 s; 10 s at 58 °C; 72 °C for 10 s with a single fluorescence measurement; a melting curve program (65–97 °C) with a heating rate of 0.11 °C/s; a continuous fluorescence measurement; and a cooling step to 40 °C. Each PCR was performed in triplicate. To determine the qPCR efficiency of each primer pair used, standard curves were generated using six serial dilutions (1:1, 1:3, 1:7, 1:15, 1:31, 1:63) of a unique cDNA sample constituted from a pool of all cDNAs obtained from each condition; qPCR efficiencies of the tested genes varied between 1.85 and 1.99. Moreover, the real-time PCR product analysis on agarose gel and by melting curve revealed a unique lane and a unique peak, respectively, indicating the formation of a single PCR product with no artefacts (data not shown). For further expression level analysis, the crossing points (CP) were determined for each transcript using the Light Cycler software. The amount of apoptotic-related genes expressed was calculated relative to the amount of the ribosomal protein F40 housekeeping gene (because of its lower coefficient of variation) using the delta-delta threshold cycle (ΔΔCt) method [59].

**Table 1:** Primers sequences for amplification, and the size of the obtained products.

Gene	Primers sequences 5'→3'	Product size (bp)	GenBank ID
Cg-FADD	AAGAGAAAGTGTCAACCGAC CTCTCAAAACATCAAGACGG	134	HQ425700
Cg-Bax like	AGGATAGCACTCTATGCAGG TCAACTCCTAGCAACCATGG	198	AM855407
Cg-Bax1	TCCACTGGAATATGTTCGAG GAAAGTTTCATGGTTTGCAC	124	HS140552
Cg-Bcl2	CAACTGTGACAAACGAGATG AGTCTACTAACTGTGGCATG	123	EU678310
Cg-BI-1	AATGGGCTTCCTGAGGAAGG GCAACCAACAGCATCCAGTG	134	HS115415
Cg-IAP1	TCGAGCAGCAATTTAACGC GAGGAAGGAGCTTTACCAC	160	HQ425702
Cg-IAP7B	CATTATGGAAGCAGATAGATC ATGATGTCATCTTCCTTTGTC	249	FP000296
Cg-caspase-2	ACAGGGGAAATACTGAAGGAC AGCTACAGCTGTCAGAAAACC	162	HQ425706
Cg-caspase-3	ATCACCAGGAAGGATCATGG GTTCATCCGAACACGACTCG	139	CU988427
Cg-caspase-7	ATTGGACCACAGAGACAACG TGTTGCCTTTGAAGGGCTCC	125	HQ425703
Cg-Hsp27	GGCAAAGACCCATTTGGTAA ACAGTCAAGTTCCGGTCCAC	206	AM862573
Cg-Hsp70	TCATCAAGTGGATGGACCAG CATTCCTCCAGGCATGCCA	149	AF144646
Ribosomal protein F40 (RPL40)	AATCTTGCACCGTCATGCAG AATCAATCTCTGCTGATCTGG	149	FP004478

### 3.6. Determination of Hemocyte Apoptosis Levels

Samples of hemolymph were collected in Modified Alsever Solution (MAS), pH 4 (v/v), then diluted in Alsever, pH 4, +8% Paraformaldehyde (v/v), and the samples were conserved at 4 °C until the analysis were performed. Single or double-stranded DNA breaks that occur at the early stages of apoptosis were detected by the red fluorescent label of DNA fragmentation (*In Situ* Cell Death Detection Kit, TetraMethylRhodamine (TMR) red, Roche^®^), according to the manufacturer’s recommendations. Briefly, hemocytes were permeabilized for 8 min. TMR red was added; then cells were incubated in the dark for 60 min at 37 °C. After three washes in phosphate buffer saline (PBS), 4′,6′-diamidino-2-phénylindole (DAPI) as added to the fixed hemocytes in the dark for 10 min. Upon staining, the fluorescent products generated by the two dyes can be visualized using a wide-field fluorescence microscope equipped with standard red (540 nm–580 nm, TMRred) and blue (358 nm–461 nm, DAPI) filter sets. The percentage of apoptotic cells was determined by counting one thousand cells from 2 × 5 individual slides by condition.

### 3.7. Statistics

Data were analyzed using two-way ANOVA followed by the Wilcoxon test (R software). Values are the median ± SD of 10 individuals from two independent experiments. * *p* < 0.01.

## 4. Conclusions

Apoptosis is highly conserved among species. In *C. gigas*, key genes involved in this process appear to be similar to those of the vertebrate model. Although Sokolova and Kiss highlighted the importance of the apoptotic process in the molluscan immune defense system [1,24], few studies have assessed the effects of harmful algae and their toxins upon the immune system of bivalves, especially on the modulation and/or regulation of the different apoptotic pathways [50]. In this study we show, for the first time, that the toxic dinoflagellate, *A. catenella*, was able to induce the apoptosis of *C. gigas* hemocytes at a toxin concentration level similar to that generally observed *in situ* during a toxic event. Since 1970, periodic and large episodes of the mortality of *C. gigas* occurred along the French coast at a level never observed before. Although several pathogens (*Vibrio splendidus*, *Vibrio aestuarianus* and Oyster Herpesvirus type 1) were demonstrated to be associated with those mortalities in the environment [58,60,61], no mortality was induced when oysters were exposed to the pathogens in our experimental conditions. It is likely that this phenomenon has a multifactorial origin. The results of this work suggest that the induction of apoptosis by PSP-producing algae may affect the efficiency of the oyster to resist microbial infection in the environment. We should further investigate this particular point.
